# Exploring the structure of household social capital in rural Vietnam: Applying Bayesian network approach

**DOI:** 10.1371/journal.pone.0305194

**Published:** 2024-07-10

**Authors:** Huynh Ngoc Chuong

**Affiliations:** 1 Faculty of Economics, University of Economics and Law, Ho Chi Minh City, Vietnam; 2 Vietnam National University, Ho Chi Minh City, Vietnam; Van Lang University: Truong Dai hoc Van Lang, VIET NAM

## Abstract

This study aims to explore the structure of the households’ social capital of rural Vietnam households with secondary data from 2008 to 2018. This paper applied the fundamental theories (resource and network theories) and the Bayesian network to estimate the interaction of proxies to explore the structure of social capital. Results showed that the triangle structure in household social capital with the core point is organization participation. The connections show the tendency from organization participation, linking to household networks. Alongside that, linking social capital and Organization participation are determinants of social capital indicators (social events, social cost). Therefore, this paper suggests employing proxies such as structured indicators for integrating social capital into the livelihood papers.

## 1. Introduction

Social capital has been a subject of considerable scholarly interest since the mid-19th century when Tocqueville made noteworthy observations regarding the significance of social gatherings and participation in American society. Scholars such as Putnam, Bourdieu, and Coleman have significantly contributed to developing social capital theory [1, 2]. Bourdieu conceptualized social capital as the accumulation of resources derived from a network of social relationships [3]. In contrast, Coleman defined social capital based on its functions, highlighting its value as a resource that individuals and organizations can utilize to attain desired outcomes. He perceived social capital as an aggregation that arises from connections and relationships among individuals and organizations [4].

Social capital is widely recognized as one of the fundamental assets that influence household livelihoods, as it fosters trust and cooperation within a community, contributing to social development and reducing transaction costs [3]. According to DFID (Department For International Development), there are five types of household livelihood assets (natural capital, physical capital, financial capital, human capital, and social capital) [5]. Moreover, social capital can replace other official institutions to ensure the trust and principles of the community, thereby promoting social development and reducing transaction costs [3, 6].

DFID (1999) emphasizes that social capital is not only a livelihood capital that promotes sustainable livelihoods but also other types of livelihood capital of the household. Nonetheless, the household-level social capital measurement still needs to be clarified and has different measurement approaches [7–9]. The relevant livelihood papers on social capital have long been a topic in livelihood choices, livelihood strategies, and most livelihood frameworks [10–12]. Research to date has simplified the quantification of social capital and used a lot of different proxies to measure household social capital [10]. Portes & Landolt (2000) stated that aggregating social capital needs to be simplified at the micro-levels, such as the total of individual social capital [13]. Woolcock & Mill figured out that measuring social capital must use a multi-dimensional approach [14].

Social capital measurement is challenging not only theoretical aspects but also practical issues. Sabatini implied that social capital is a differential formation regarding space-time of studies [15]. According to Yobe et al. (2019), the authors applied the resource theory to collect the number of information sources for measuring social capital [16]. Wang et al. (2019) aggregated social networks to present the social capital of households [17]. Social capital is viewed to have a dynamic, complicated, and interdisciplinary nature. Therefore, it is challenging to measure whatever in levels [18–21]. In the livelihood literature, many proxies is used to indicate the household social capital such as organization participation, household network, information sources, or market access frequency [10]. In Vietnam, the role of social capital is becoming more and more important for enhancing households’ livelihood. Through social links, households can access resources or economic benefits such as supports, financial access or new information, especially in natural disasters [22, 23].

Literature review shows that Vietnam has many differential dimensions of household social capital. Some studies have applied qualitative methods to explore these characteristics, pointing out the differences between Vietnam and other countries. Other studies have focused on various dimensions of social capital, elucidating the distinct characteristics of social capital in Vietnam [24–26]. Chuong and Chi Hai employed the MIMIC approach to explore the structure of social capital. This paper relies on the MIMIC approach to examine the established theoretical framework [9]. Therefore, more research is needed to explore the structure of social capital in Vietnam from different perspectives.

Bayesian Networks are graphical models encompass information about the causal probabilistic relationship between variables [27]. These probabilistic causal relationships in the network can not only “learn to investigate” significant relationships but also continuously update as new information to enhance the identified results. Some studies applied the BN not only to structure the indicators but also to illustrate their relationships [28–30]. Kemp-Benedict et al. applied the BN to assess the relationships between household capitals as well as household livelihood aspects [31]. Meanwhile, Junquera and Grêt-Reganmey assessed the livelihood vulnerability using the BN model, they showed that the BN model reflects and allows investigation of the dependence relationships between indicators [32]. Other studies confirmed that using the BN approach usefully elucidates the dependence relationships or recognizes the interdependencies between various variables [32, 33]. Therefore, the BN approach is suitable for investigating vague structures such as social capital.

This study aims to explore the households’ social capital structure by applying an integrated framework of the theoretical aspects. This paper combines the indicators of social capital aspects that are based on theories as well as empirical studies. In addition, the authors investigate the relationships among social capital components by using the Bayesian network approach. The authors expect to confirm its direct and indirect relationships. Therefore, this paper contributes to the literature in 2 aspects: first, this paper applied an integrated theoretical approach by collecting and aggregating social capital proxies to explore the structure of social capital at the household level; second, with our knowledge, this paper is the first paper applying the bayesian network to estimate and visualize the network of social capital proxies which helpful depict the structure of household social capital.

The paper’s outline is as follows: Section 2 describes the theoretical view of social capital and the livelihood view of social capital proxies at the household level. In section 2, we focus on synthesizing theoretical social capital views and livelihood views to enhance the household social capital indexes for household livelihood research. Section 3 describes the data set and the Bayesian network approach to estimate the conceptual framework in the third part. Section 4 illustrates an overview of household social capital in rural Vietnam and then discusses the model results. In section 5, this paper implies some recommendations for measuring household capital for livelihood research.

## 2. Theorical approaches in social capital views and livelihood views

### 2.1. From concepts to formations

Social capital was noticed in the mid-19th century with Tocqueville’s view of meeting and participation in American society. Putnam, Bourdieu, and Coleman contributed to the milestone of social capital views [1, 2]. Social capital under Bourdieu’s view, social capital is institutionalized from a network of acquaintances that create the existing or potential resources (Portes, 1998). In the view of Coleman: "Social capital is defined by its functions", social capital is different from other types of capital; social capital is not tangible but is the accumulation through connections or relationships between individuals and organizations. Coleman identified it as an active resource consisting of three forms: negotiation and expectation, information, and social norms [34]. Numerous researchers have further developed and examined the theory of resource-based social capital [3, 13, 35].

According to Fukuyama, social capital cannot be an individual activity, and it creates and transmits cultural elements such as traditions, religions, historical habits, and common goals [36]. Meanwhile, according to Wilson, social capital is a self-organizing system with many stakeholders connected to the community [37]. According to Nahapiet & Ghoshal stated that social capital is the aggregation of real and potential resources rooted in individuals’ networks [38]. Portes defined social capital as (1) a resource controlled by society, (2) a resource of family benefits, and (3) resources through non-family networks [3]. On the other view, Fukuyama states that social capital is connections through social relationships that foster trust and cooperation. Therefore, connections are the core elements in social capital, helping to reduce transaction costs in economic activities or supporting the success of a democratic society [36].

Lin develops social capital concepts based on the literature on networks and social capital. Lin argued the position of social capital is identified in social networks or through investment in social capital networks or access to social resources to achieve a value like financial benefit [39, 40]. Moreover, Lin asserted that a higher level of social position in the social capital network achieves a higher level of benefit return. Meanwhile, Woolcock & Narayan reinforces the concept of social capital from the perspective of the object of research, which separates into four perspectives: the public view, institutional view, network perspective, and integrated perspective [41].

Portes also identified four potential adverse effects of social capital, including those outside the network, organizational rules and requirements, limitations on personal freedom, and the dark sides of norms [3, 13, 35]. The utilization of social capital resources is widely employed in livelihood approaches, with the Department for International Development [5] defining social capital as the social resources people rely on to pursue livelihood goals. Many studies on household livelihoods and livelihood frameworks adopt the perspective of social capital as a resource, treating it as one of several capital types (natural, physical, financial, and human capital) exploited to achieve livelihood objectives [5, 42, 43]. Thus, regarding social capital resources, the exploitation of social resources and access to resources through household networks can be utilized to pursue livelihood strategies.

From a network perspective, the connections between individuals, groups, and organizations serve as the core elements of social capital. Putnam highlighted the role of organizational membership in social activities and economic benefits [34, 44]. Putnam primarily focused on formal links among individuals. In the view of Putnam, social capital is considered from a macro perspective in aspects: norms, trust, or social values. Putnam also made significant contributions to social capital theories, particularly in the dimension of social resources, where social norms and trust serve as core elements for leveraging social capital resources. Additionally, Granovetter and Burt developed the theories of weak ties and structural holes, respectively. Granovetter proposed the theory of weak ties, which suggests that social capital is not only present in solid or formal relationships but also in weak ties within networks. Weak ties give individuals access to new information and more significant opportunities [45].

Burt’s theory of structural holes, presented in his books "Structural Holes" and "Brokerage and Closure" [46, 47], deepens the understanding of individual connections within networks and social structure. In this network perspective of social capital, the theory of structural holes delves into four patterns: contagion, prominence, closure, and brokerage, supported by three main empirical pieces of evidence: the link between rewards and performance mediated by social capital, evidence of creativity and learning. Thus, the development of social capital theories reveals that the characteristics of household capital lie within relationships, including strong ties, weak ties, and structural holes. Within the complexity of social networks, households can benefit from opportunities or information by leveraging structural gaps.

From a theoretical and empirical standpoint, Woolcock and Narayan and others emphasized the "dynamic" nature of social capital, which consists of three forms: bonding, bridging, and linking, all of which contribute to social cohesion and are prevalent in general social capital patterns [41, 48–50]. Specifically, social capital comprises three components: (i) bonding social capital, which encompasses direct relationships such as family, friends, and neighbours and is based on strong interpersonal relationships and direct interactions; (ii) bridging social capital, which includes more distant relationships such as colleagues, organizations, and homogenous connections; (iii) linking social capital, which involves connections to individuals with hierarchical links.

### 2.2. Empirical proxies and conceptual framework

Livelihood studies draw upon theoretical frameworks such as sustainable livelihood analysis [5, 43, 51]. DFID framework serves as a fundamental theoretical foundation for livelihood studies, viewing household livelihood capitals as the determining factors for livelihood strategy decisions. The framework identifies five core capitals: human capital, social capital, natural capital, physical capital, and financial capital. Within this framework, social capital plays a significant role, serving as a source of livelihood assets and influencing other forms of capital. Social capital is discussed regarding networks, organizations, trust, and exchange. Consequently, many livelihood papers emphasize social capital as a critical factor within various livelihood frameworks and models. However, the indicators used to measure social capital differ across the literature. Some papers employ relationship indicators such as the number of close friends, neighbours, relatives, and friends to assess household social capital [52–55], focusing on the network aspect of social capital. These studies consider social capital as an element of social relations that can be leveraged to pursue household livelihoods.

Furthermore, studies often utilize participation indicators in different organizations along with political linking [56–58]. In these cases, the resource aspect of social capital and social capital linking are applied [59–61]. Other researchers use various indicators to express social capital, such as grants, social costs, gifts, or participation in social activities like meetings and festivals [54, 62–65]. These approaches employ the household’s social capital output as an index to measure social capital. Moreover, various indicators such as the frequency of shopping [52], the number of visits [56], trust in neighbours [64], or cellphone usage [66] are employed in different livelihood estimation models to represent social capital. Social activities and social costs indicate household social capital outputs, revealing the social characteristics of household networks and relationships [59, 62]. Additionally, social costs are used to maintain connections and strengthen social relationships in Vietnam [63].

**Fig 1:**
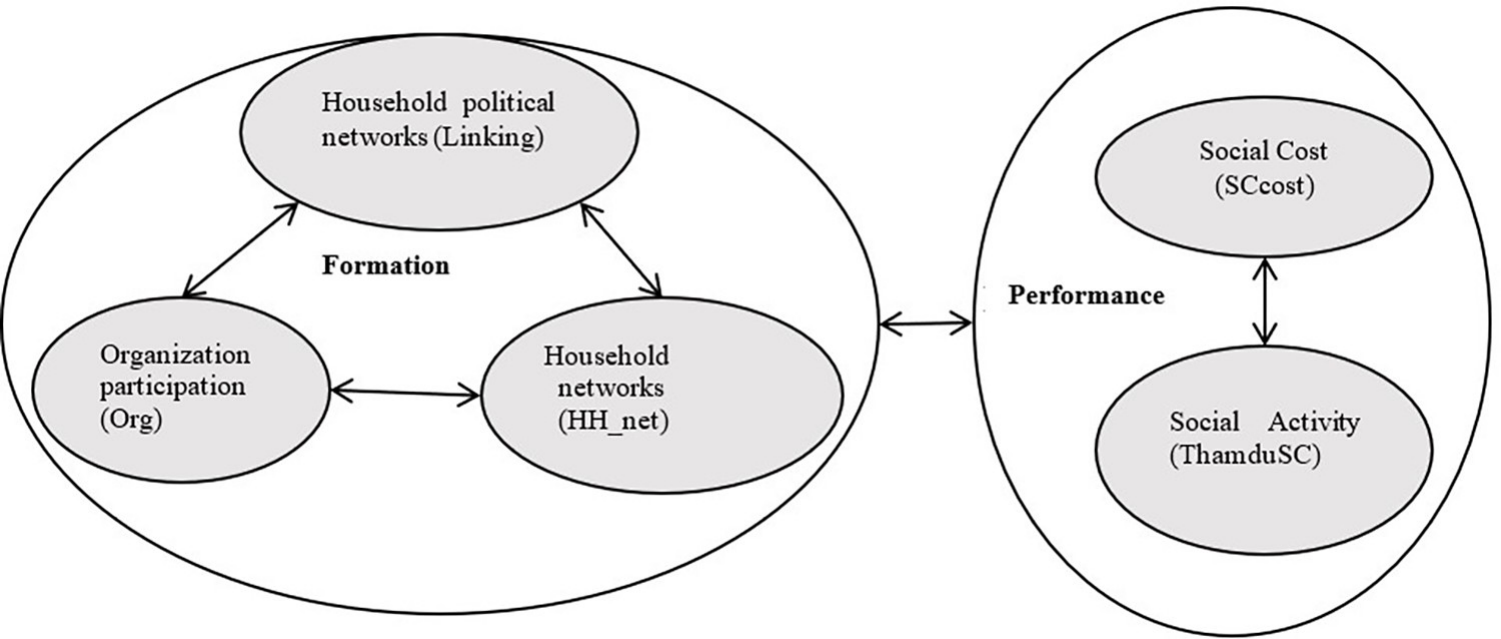
The paper’s conceptual framework.

This paper proposes a new approach (Fig 1) to exploring the interaction among social capital indicators. The proposed framework has two pillars: firstly, the formation of social capital structure or structure social capital based on the social capital theories. The dynamic approach from Woolcock and Narayan stated that social capital structure has 3 aspects: bonding, bridging, and linking capital [41]. The bonding-bridging social capital is measured by the degree of hierarchical linking within the organizations where households participate. Meanwhile, the linking social capital of households is accounted for by the degree of connection to public/ government organizations or political person. Meanwhile, the performance of social capital at household level is used to indicate social activities of households [67, 68]. Two indicators will be used: participation in social events (ThamduSC) and social costs of the household (SCcost).

We hypothesize the links between the formations and the performance of social capital at the household level. Through the conceptual framework, this paper will investigate to elucidate not only the connection between formation and performance of household social capital but also delving into interaction between indicators of social capital.

## 3. Data and econometric approach

### 3.1 Data

This study uses VARHS (Vietnam Access to Resources Household Survey) dataset in 2008 and 2018 in 12 provinces in Vietnam, including Hanoi (a part of Hanoi–formerly known as Ha Tay), Nghe An, Khanh Hoa, Lam Dong, Dak Lak, Dak Nong, Lao Cai, Dien Bien, Lai Chau, Phu Tho, Quang Nam, and Long An [26]. In this study, the author filtered missing observation of dataset. R software is utilized for data manipulation and econometric model estimation. We use the “bnlearn” package for estimating Bayes Network.

The formation part is that the three forms of social capital determine the nature of household social capital [41, 49, 50]. This paper depends on the households’ connections with organizations and social networks to measure these three forms of social capital. The bonding-bridging social capital is measured by the degree of hierarchical linking within the organizations where households participate. Meanwhile, the linking social capital of households is accounted for by the degree of connection to public/ government organizations or political person. Therefore, three indicators are used: the number of Organization participation (Org), amount of household networks (HH_net), and amount of household political networks (Linking). Besides, two indicators are used to indicate the performance of household social capital: participation in social events (ThamduSC) and social costs of the household (SCcost).

Therefore, we extract five variables from the VARHS dataset: the number of Organization participation (Org), amount of household networks (HH_net), amount of household political networks (Linking), participation in social events (ThamduSC) and social costs of the household (SCcost). All variables are listed in Table 1.

**Table 1:** The number of household networks and linking connections.

Component	Variable	Measurement	Previous studies
Formation	Organization participation	the number of Organization participation (Org)	[41, 49, 50]
	Household political networks	amount of household political networks (Linking)	
	Household networks	amount of household networks (HH_net)	
Performance	Social Cost	Total Social Cost (SCcost)	[67, 68]
	Social Activity	Amount of household’s social activites (ThamduSC)	

### 3.2 Bayesian network

The author proposes using Bayesian Networks (BN) to explore the structure of social capital among rural households in Vietnam. Bayesian Networks are part of the graphical model framework, often called Directed Acyclic Graphs (DAGs), representing conditional independence relationships between variables. BN models are also known as Belief Networks or Bayes Nets [69].

The dependency structure between variables is represented by nodes (variables) and directed arcs in the DAG, indicating conditional relationships. Therefore, the joint probability distribution of variables X1, X2,…, Xn can be factorized as follows:

$$P(X1,X2,..,Xn)=\prod_{i=1}^{n}P(X_{i}|\prod_{i})$$

Where ∏_*i*_ is the parent set of Xi, and P represents the joint probability mass or density function when Xi is discrete or continuous, respectively.

The advantage of the BN method is that it provides an intuitive understanding of complex relationships between variables, particularly the nonlinear relationships between dependent and independent variables and among independent variables. BN can be used as a principled, nonparametric framework for causal inference [27, 70]. Ultimately, BNs can be used as a structured instrument to learn about the interaction among the indicators. Mixed algorithms offer both statistical evidence and sensitivity in assessing the strength of relationships between nodes (variables) and the robustness of the BN model.

Based on the mixed approach, this paper identifies the BN model or network of relationships, interactions, and sensitivity effects. The optimized Bayesian network model estimation based on the two-phase hybrid approach (employing Semi-Interleaved HITON-PC with Pearson correlation and a significance level of 5%, combined with the Bayesian point estimation utilizing Hill-Climbing) yielded results from various tests [71]. In addition, this paper performs a sensitivity assessment in the interaction of variables which transfers their values to four categories: low (L), medium (M), high (H), and very high (VH). The Bayesian network model, based on probability variation grouping of nodes (variables) and conditional probability table (CPT), provides insights into the varying states of the nodes and the sensitivity in the relationships between different forms of social capital.

## 4. Estimated results and discussion

### 4.1 Overview of organization participation in Vietnam

Table 2 indicates that most households participate in at least one organization. The survey results reveal that all households are engaged in at least one social organization. Moreover, the more organizations in which households participate, the more their network expands. The highest number of household members participating in any organization is 10, and the most common scenario is households participating in two organizations, accounting for over 37% of the surveyed households.

**Table 2:** The number of household members participating in organizations.

Member	Year						Total (%)
	2008	2010	2012	2014	2016	2018	
1	27.27	32.65	26.24	28.45	32.14	30.44	29.55
2	41.98	34.64	37.93	35.89	37.60	36.52	37.37
3	16.16	16.97	20.24	20.72	17.77	19.12	18.54
4	8.46	9.69	10.24	9.22	8.27	9.32	9.21
5	3.66	3.35	3.43	3.21	2.40	3.42	3.24
6	1.45	1.47	1.16	1.26	1.29	0.94	1.26
7	0.57	0.70	0.58	0.86	0.23	0.18	0.52
8	0.38	0.41	0.12	0.11	0.06	0.06	0.19
9	0.06	0.12	0.00	0.17	0.23	0.00	0.10
10	0.00	0.00	0.06	0.11	0.00	0.00	0.03
Anova Test	100.00	100.00	100.00	100.00	100.00	100.00	100.00

Note: percentage households in unit.

Source: Calculation from dataset

At the household level, the average overall network connectivity over the years indicates an expansion of households’ network connections. That is a positive trend regarding the benefits derived from social networks. In 2008, the average network connectivity per capita for households reached a level of 0.56, gradually increasing over the years, and by 2018, the average per capita network connectivity reached 0.67. The results show a significant increase in the scale of organizational involvement of households in Vietnam’s rural areas.

### 4.2 The role of organizational participation

Among the surveyed households over 12 years, there has been a substantial shift in the perceived importance of organizations for family households. Table 3 results reveal that households not perceiving any organization as important decreased from 6.2% in 2008 to 4.72% in 2018. The number of households confirming the significant influence of organizations on their well-being (3 households) experienced a significant increase from 37.44% to over 50% of the responding households. These results demonstrate the significant role of organizations in the overall socio-economic livelihoods of households and their specific income-generating activities.

**Table 3:** The important organizations of households.

Organizations	Year						Total
	2008	2010	2012	2014	2016	2018	
0	6.12	4.99	8.78	5.32	5.10	4.72	5.84
1	28.35	35.06	25.25	22.04	27.68	21.18	26.55
2	28.09	20.96	19.66	22.10	25.40	23.30	23.19
3	37.44	38.99	46.31	50.54	41.82	50.80	44.42

Note: percentage households in unit.

Source: Calculation from dataset

Among the organizations that play an essential role for households, statistical results indicate that only about 35 households in 2018, out of 192 households with party members, do not consider the Party organization as one of the top 3 crucial organizations for their households. Most households with party members (approximately 81.77%) perceive the Party organization as crucial for their households. Regarding political and social organizations, households value the importance of these groups. On the other hand, despite high levels of participation in voluntary social organizations or economic cooperative organizations, the proportion of households whose members perceive them as less important is much lower compared to political organizations.

### 4.3 Households networks

Table 4 results show that the proportion of household members participating in public organizations in 2018 is less than 6%. Regarding the household’s connection to public organizations through relatives or extended family, the statistics for 2018 reveal that approximately 18% of households have at least one relative working in public organizations, indicating a significant increase compared to the 5% in 2008. Furthermore, the household’s connection to public organizations through close friends is quite high, with around 24% of households having at least one close friend working in public organizations. Compared to 2008, there has been a significant increase in connections to public organizations through friends (only about 5% in 2008), indicating a substantial increase in network connections.

**Table 4:** The number of household networks and linking connections.

Year	The number of connections							Average number
	0	1	2	3	4	5	6	
2008	94.66	0	0	0	1.01	4.03	0.3	0.26
2010	94.36	0	0	0	1.86	3.32	0.45	0.27
2012	95.72	0	0	0	0.6	3.07	0.6	0.21
2014	94.51	0	0	1.51	2.17	1.71	0.1	0.22
2016	66.77	8.11	16.16	4.53	3.93	0.35	0.15	0.72
2018	65.84	8.76	14.31	8.61	2.23	0.26	0	0.73

Note: percentage households in unit.

Source: Calculation from dataset

The linking connectivity demonstrates significant differences among households. The average linking connectivity has significantly increased from 0.26 in 2008 to 0.73 in 2018, with a corresponding increase in the percentage of households, from approximately 6% in 2008 to approximately 35% in 2018.

### 4.4 Social activity and cost

Table 5 shows the frequency of participation in social activities. In 2008, each household participated in about 17 social events, which remained similar based on the 2018 survey (with an estimated average of 16 events). Over the years, half of the surveyed households consistently attended an average of one event per month (15 events per year). The total costs of attending these events varied widely, ranging from inexpensive to very high expenses.

**Table 5:** Social cost.

Year	Min	Average	Median	Max	SD
2008	30	9613.485	8000	65000	10703.76
2010	20	12637.54	12000	50000	11839.14
2012	50	22347.06	15000	120000	25194.49
2014	40	17467.54	5000	123000	25427.51
2016	40	4561.176	300	70000	16615.34
2018	100	33785.51	15000	350000	53441.28

Note: thousand Vietnam Dong (1000 VND) unit, 1 USD ≈ 25000 VND (exchange rate in 2024)

Source: Calculation from dataset

In 2008, the highest recorded total cost for attending events was 60.4 million VND; in 2018, it peaked at 300 million VND. However, overall, the total costs of household participation increased significantly. Around 50% of the households had total expenses below 50,000 VND in 2008, but this figure gradually increased over the survey years. By 2014, 2016, and 2018, the expenses reached approximately 200,000 VND per year, quadrupling the previous amount. Additionally, the average cost per social activity also increased over the years. In 2018, 50% of the households reported an average expenditure of 15,000 VND per event, three times higher than in 2008.

Furthermore, alongside participation in social activities, gift-giving and receiving play an important role in Vietnamese society, particularly in traditional East Asian societies. It is a form of social relationship expression during festive occasions and a means of building social connections in Vietnam. Through surveys conducted among rural households in Vietnam, the total value of gifts given and received exhibited significant fluctuations. The lowest recorded value for received gifts was 30,000 VND in 2008, with an average of approximately 9 million VND. However, in 2018, the lowest recorded value for received gifts was 100,000 VND, averaging 350 million VND.

Furthermore, there was a significant disparity in the value of gifts exchanged among households. The total value of gifts sent and received exhibited considerable variation, with the lowest recorded value for sent gifts at 2,000 VND in 2008 and an average of approximately 65,000 VND. In contrast, in 2018, the lowest recorded value for sent gifts was 50,000 VND, with an average exceeding 3 million VND.

### 4.5 Bayesian network results and discussion

#### 4.5.1 Bayesian network estimation

**Fig 2:**
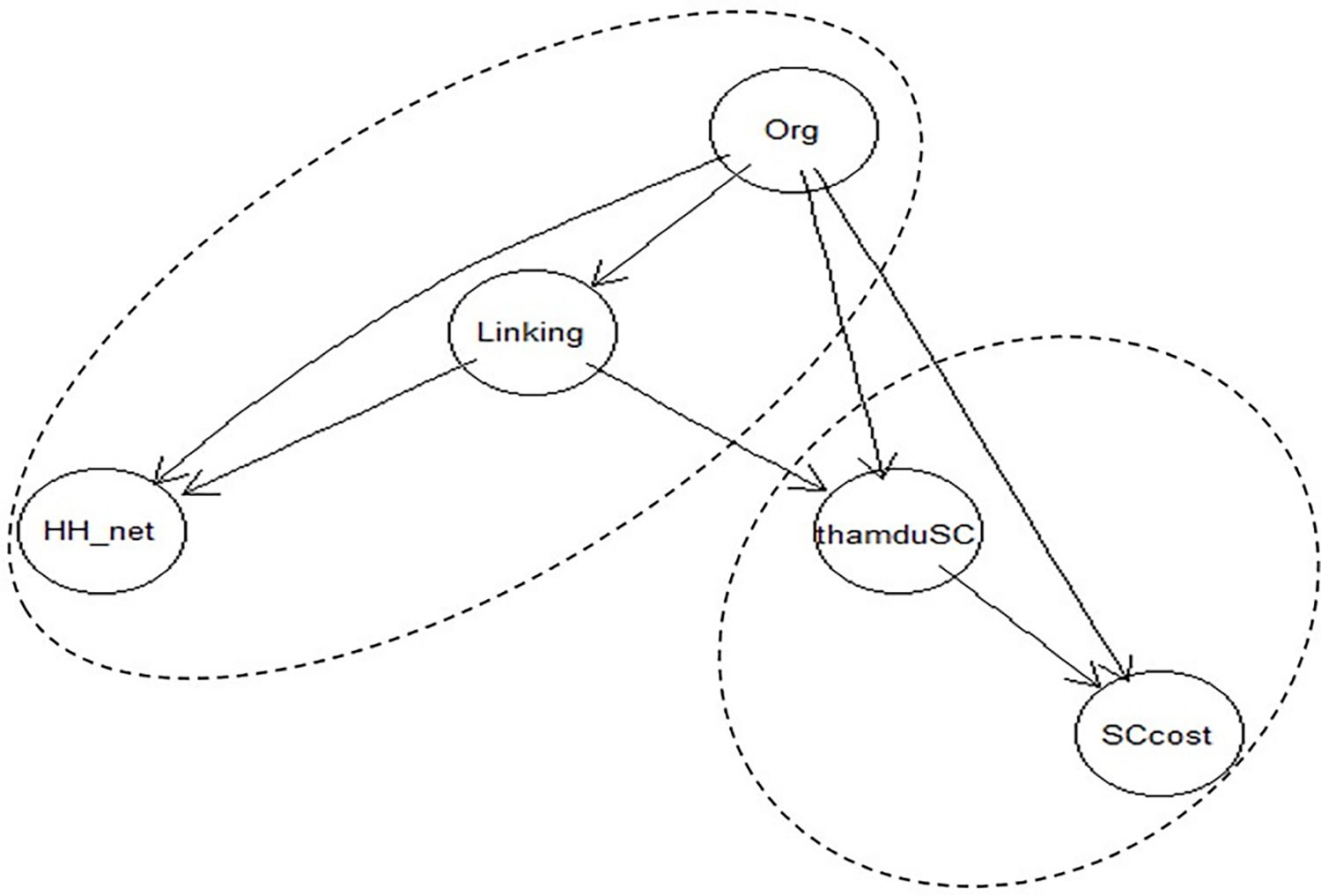
Result of the Bayesian network estimation.

The Fig 2 results of the social capital network mode identified seven statistically significant relationships based on the research data. All seven relationships were unidirectional, with the variable "Org" (number of participating organizations) representing the starting points of the relationships. Conversely, the "HH_net" node (household social network) and "Sccost" node (social costs of the household) were outcome variables influenced by other variables.

The interactions between forms of social capital within households, as determined by the Bayesian network model. The formation of social capital in Vietnam households shapes the triangle structure. The central pillar is participation in organizations (Org). It directly affects two other forms (Linking and Household networks). In addition, “Linking” (linking connections/networks) also affects Household Networks (HH_net). The relationship between “Formation” and “Performance” through 3 channels: 2 channels from “Org” (Organization Participation) to Social Activities (thamduSC) and Social Cost (SCcost), one channel from Linking social capital (Linking) to Social Activities (thamduSC).

#### 4.5.2 The formation of household social capital

Regarding the impact of the number of organizations in which households participate, increasing the number of participating organizations enhances the level of engagement in social events/activities (thamduSC), as well as the social network (HH_net) and the linking connectivity (Linking). However, the results also indicate that increased participation in organizations tends to decrease the social costs of households with social engagement.

On the other hand, for the form of social capital based on the household’s networks, an increase in participation in organizations and the degree of household connectivity also lead to an increase in this form of social capital. Specifically, the direct impact from the degree of household connectivity is approximately twice as strong as the direct impact from participation in organizations. Nevertheless, participating in organizations also indirectly influences the household social network, resulting in a slight reduction in the overall impact of the organizational variable, although still with a noticeable negative effect.

Regarding the form of social capital known as degree linkage, the model’s results indicate that household connectivity is significantly influenced by participation in organizations. This impact is the highest among all the relationships considered in the Bayesian network model. This result confirms the critical role of participating in organizations for the degree of linkage social capital of households. Additionally, the social capital Linking (Linking) directly impacts the household social network, significantly increasing it, with the second highest impact after the relationship between organizations and the degree linkage of households. Furthermore, the degree linkage of households has a minor indirect impact (the lowest among all the impacts) on the social capital costs (SCcost).

#### 4.5.3 Sensitivity of the structure of households social capital

**Fig 3:**
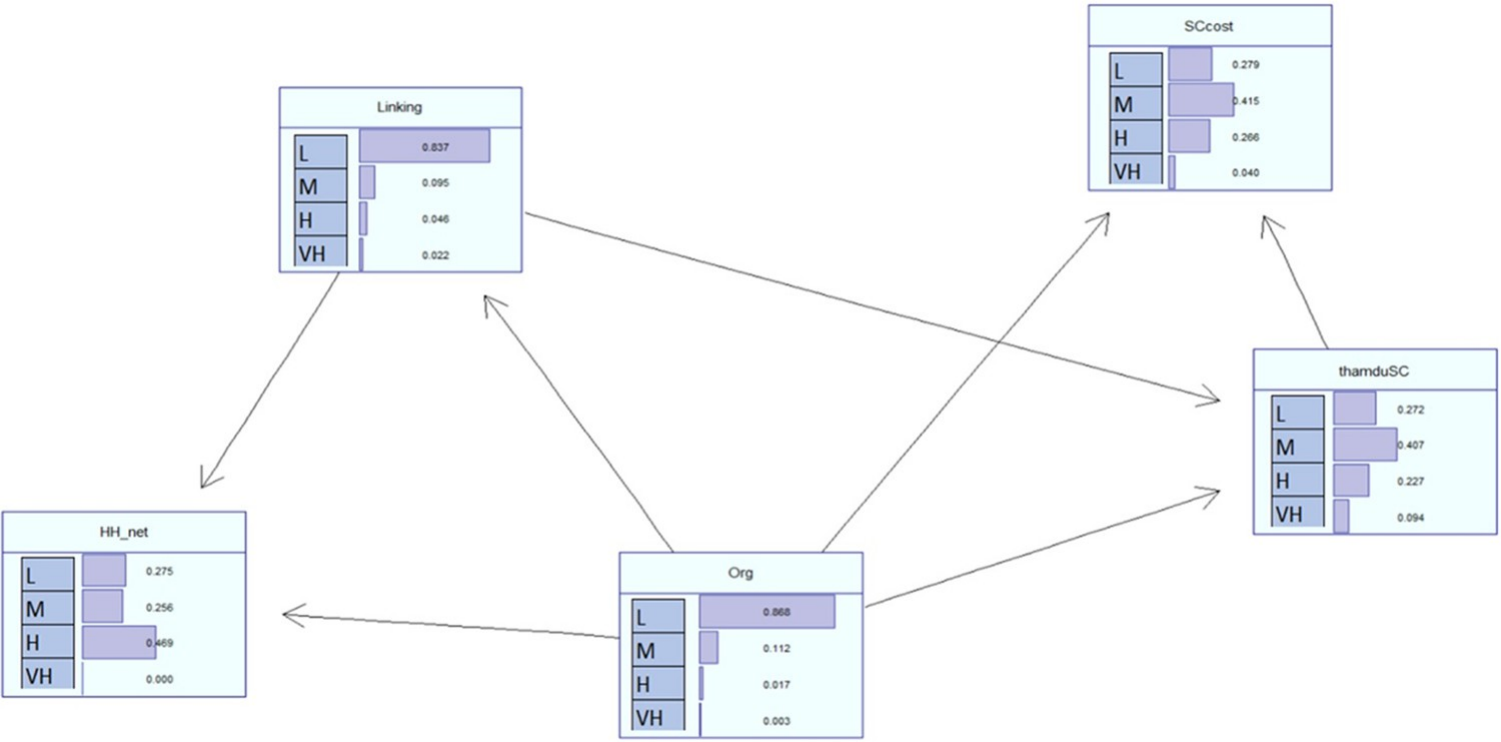
The sensitivity of model interaction. Source: Model estimation.

The probability distribution of the levels in the forms of social capital reveals significant differences in Fig 3. The relationship between organization participation (Org) and Linking (Linking) is primarily concentrated in the low level (with approximately 83.7% to 87% of households falling into this category) and decreases sharply in higher categories. Specifically, only 0.3% of households have a high level of participation in organizations, and 2.2% have a high degree of Social Capital Linking (Linking).

In contrast, the household social network (HH_net) demonstrates a broader distribution, with most households reaching medium and high levels, with nearly half of the households classified as high. As for social activities and social costs, the probability distribution shows relatively similar patterns in the low and medium levels, which are the two predominant categories. However, the distribution of probabilities for the high and very high categories in social activities and social costs is significantly higher than the participation in organizations and the degree of social capital linking (Linking).

Low-level participation in organizations (Org) strongly influences the degree of social capital linking (Linking) and significantly impacts the household social network. The estimated highest probability is that the household will be in a state with low-level “Linking” and a moderate network (HH_net), with a probability of 36.4%. The second most likely scenario is that the household will be in a low-level state in both Linking and HH_net, with a probability of approximately 25.8%.

The social activity level (thamduSC) of Vietnamese households shows that social activities are directly influenced by participation in household organizations (Org) and Linking connections (Linking). Regarding the social cost (SCcost), it demonstrates a partial impact of participation in organizations (Org) and engagement in social activities (thamduSC) on the social costs of households. The influence of low organizational participation on the social costs (SCcost) at a low state primarily indicates a high probability (over 85%). However, there is still a relatively high probability of social activities at an average level (13%). On the other hand, as the household’s level of participation decreases, the probability of the household incurring higher social costs tends to increase. However, this is only true for participation levels from low to moderate. At a high level of organizational participation, high and moderate social costs are possible outcomes.

### 4.6 Discussion

The household social capital is constructed and vary by context [5, 72]. The assessment of the social capital model of Vietnamese households reveals that the formation of household social capital is context dependent. The index of organizational participation is a major contributor to the social capital of households in Vietnam. The Bayesian network model demonstrates the relationship between different forms of social capital and their connection to social indicators (“performance”).

In terms of the relationship between different forms of social capital, the three forms exhibit a triangular connection pattern. The factor of participation in political and social organizations has a significant influence on the households’ Linking (Linking) and the social network of households (HH_Network). The degree of Linking also affects the households’ networks. In other words, the capacity within the household’s social network is influenced by its participation in organizations and the degree of linking connectivity. In Vietnam, households gain easy access to information if their family members participate in core organizations such as the Party, Youth Union, Women’s Union, Veteran Union, and Farmers’ Union. Members of these unions not only have advantages in accessing useful information but also receive priority access to livelihood resources. The network aspect also contributes to the formation of household social capital, albeit with a lower but unquestionable effect. It enhances the scale of household social capital, especially in terms of network connections. Social capital derived from network connections plays an important role in supporting disaster recovery or coping with livelihood shocks [73–75]. On the other hand, the network sides contribute to the formation of household social capital on the lower but unquestionably effects. Its effects enhance the scale of household social capital, especially in the network linking. From the viewpoint of livelihood papers, social capital rooted from network side plays a important role of support for disaster recovery or livelihood shock context [76–78]. The characteristics of organizations generate the scale of household social capital in the bridging and linking dimension. Households can exploit that face of social capital like a mystery resource in the livelihood view. In which, linking connections is of great source for recovery pathway of household and bonding is directly maintain the food insecurity in the shock context, particularly in rural regions and the poorest households [79]. Households can leverage these aspects of social capital as valuable resources in their livelihood strategies. Linking connections provide a source for the recovery pathway of households, while network bonding directly affects food insecurity in shock contexts, particularly in rural regions and the poorest households [73, 80]. In Vietnam, households’ closed networks often consist of relatives, leading them to rely more on informal channels than formal ones. Therefore, the contribution of the network aspect may not be fully observed [81].

Regarding the relationship between different forms of social capital and social indicators of households, there is a unidirectional relationship from social capital forms to the level of social indicators. Participation in organizations influences both aspects of social capital, including the level of participation in social activities and social costs. However, the degree of linking connectivity only affects the level of participation in social activities without influencing social costs. Furthermore, the social network aspect of households does not provide sufficient statistical evidence to confirm its impact on social indicators.

Furthermore, the social costs households incur are significantly influenced by their level of participation in social activities. Through this relationship, we can ascertain the indirect impact of the degree of hierarchical connectivity and the extent of engagement with organizations on the social costs borne by households. In order to maintain social connections, households must participate in various social activities, particularly during traditional festivals, village events, and similar occasions. A larger network implies that a household has numerous connections and contacts. Within the framework of traditional Vietnamese culture, receiving an invitation to partake in a traditional festival, village gathering, or neighbourhood event is considered a sign of respect [74, 75]. During these events, households can connect with individuals from higher social strata and expand their networks. Therefore, engaging in social activities demonstrates respect towards the hosts and signifies the social standing of the household and its members. Social activities encompass active involvement in these associations and social networks, which are closely associated with the subjective aspects of social capital (Pileček et al., 2013 [68]).

A high level of household participation in social activities reflects high social capital. Frequent engagement in social activities demonstrates the maintenance and representation of social connections within social capital. A high level of social capital implies that the household has significant social connections, thus making social activities an important indicator of this connectivity. Furthermore, participating in social activities helps households maintain existing connections and creates opportunities to establish new social networks [82]. Additionally, as social capital increases, the costs associated with organizations, groups, and community involvement that households bear also increase. The results represent a cost similar to the maintenance expenses of social capital. However, social costs and gift costs differ in terms of implementation. Social costs are related to collective activities, groups, and organizations in which household members participate, while gift costs serve to maintain and expand households’ social networks [83].

Consequently, gift-giving has become a prominent cultural characteristic in Vietnam, where households and individuals often associate gifts with cultural customs and social interactions. By giving gifts, households maintain their network connections and establish social positions within the community. This characteristic gradually diminishes when considering the community’s expectations of its members in Vietnam. One of the social expectations is the reciprocal contribution of community members, aimed at expressing the bonds between individuals within the community [84, 85]. This notion aligns with the common understanding of trust and long-term cycles, as suggested by [37, 86].

## 5. Conclusion

*Social capital* is a multifaceted construct that cannot be viewed as a homogeneous dimension or a singular index. This paper is the first attempt to illustrate the interaction of the social capital indicators at the household level. The BN approach well depicts the probability relationships among the different dimensions of social capital. By visualizing all directional relationships among various indicators, this paper has shown the map of dependencies and interactions among all aspects of social capital at the household level. From the conceptual framework, we focused on the structural learning algorithm used to estimate the graphical structure of households’ social capital. The estimation results confirm the triangular connection pattern of social capital formation of households in Vietnam’s rural areas. In addition, organization participation and linking connect to the social activities and social costs in Vietnam.

This paper suggests some implications in practical policy and further research of household’s social capital. Firstly, social capital has many components that exhibit interactive relationships; therefore, policies should target specific dimentions to influence households’ social capital, especially in organization participation. Secondly, policies can encourage households to engage in community activity as well as social organizations to enhance their social capital. Finally, further livelihood research can integrate the structure of social capital to analyze the role of social capital dimensions on household outcomes. In Vietnam, researchers can consider using organizational indicators to measure household capital formation or employing social activities to reflect household social capital. In general, this methodology could be suitable for exploring insights into the multifaceted concepts and complementary existing livelihood analysis.
